# 6D Object Pose Estimation Based on Cross-Modality Feature Fusion

**DOI:** 10.3390/s23198088

**Published:** 2023-09-26

**Authors:** Meng Jiang, Liming Zhang, Xiaohua Wang, Shuang Li, Yijie Jiao

**Affiliations:** School of Electronic Information, Xi’an Polytechnic University, Xi’an 710048, China; m13259460281@163.com (M.J.); zhanglm200005@163.com (L.Z.); lishuang2022918@163.com (S.L.); jiaoyijie2022@163.com (Y.J.)

**Keywords:** 6D pose estimation, RGB and depth modality fusion, attention mechanism

## Abstract

The 6D pose estimation using RGBD images plays a pivotal role in robotics applications. At present, after obtaining the RGB and depth modality information, most methods directly concatenate them without considering information interactions. This leads to the low accuracy of 6D pose estimation in occlusion and illumination changes. To solve this problem, we propose a new method to fuse RGB and depth modality features. Our method effectively uses individual information contained within each RGBD image modality and fully integrates cross-modality interactive information. Specifically, we transform depth images into point clouds, applying the PointNet++ network to extract point cloud features; RGB image features are extracted by CNNs and attention mechanisms are added to obtain context information within the single modality; then, we propose a cross-modality feature fusion module (CFFM) to obtain the cross-modality information, and introduce a feature contribution weight training module (CWTM) to allocate the different contributions of the two modalities to the target task. Finally, the result of 6D object pose estimation is obtained by the final cross-modality fusion feature. By enabling information interactions within and between modalities, the integration of the two modalities is maximized. Furthermore, considering the contribution of each modality enhances the overall robustness of the model. Our experiments indicate that the accuracy rate of our method on the LineMOD dataset can reach 96.9%, on average, using the ADD (-S) metric, while on the YCB-Video dataset, it can reach 94.7% using the ADD-S AUC metric and 96.5% using the ADD-S score (<2 cm) metric.

## 1. Introduction

The 6D object pose estimation plays a pivotal role in various domains, such as machine vision [1], augmented reality, virtual reality [2,3], motion tracking [4], and robotic grasping [5,6,7]. Vision sensors capture input images, and additive manufacturing processes are great techniques used to produce sensors [8,9]. The 6D pose estimation results are influenced by input data. When dealing with a single RGB image input [10,11], the process of estimating 6D pose heavily relies on the presence of abundant texture information. On the other hand, when utilizing a point cloud generated from depth images [12,13], it becomes crucial to have an adequate amount of geometric information. However, in complex environments, methods relying solely on modalities tend to reduce the accuracy of estimation. Therefore, the simultaneous use of both modalities is vital for 6D object pose estimation.

Many studies have considered combining information from RGB and depth image data sources. Traditional methods [1,14,15] extract descriptors from both RGB and depth images. They utilize the extracted descriptors to compute the similarities or distances between the query descriptors and reference descriptors. Finally, the data from both RGB and depth modalities are combined to determine the final pose estimation. These methods alleviate the problems associated with single-modality methods by detecting key points on low-texture objects. However, due to the limitations of manual feature extraction and fixation matching processes, it is difficult to resist occlusion with such methods.

**Figure 1:**
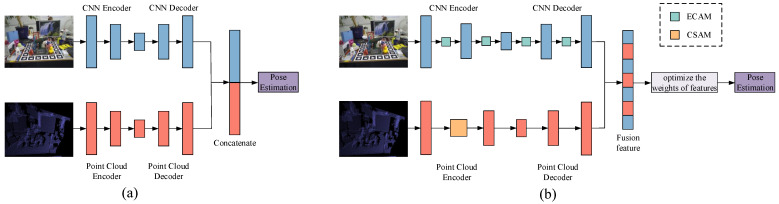
Network comparison. (**a**) The DenseFusion [22] network. The features of the two modalities are directly concatenated. (**b**) The proposed fusion network. It considers the interactive information of a single modality and cross-modality for enhanced appearance and geometry information.

In recent years, the rapid development of deep learning technology has sparked considerable research [16], and it is very important to extract and select features suitable for the task [17]. Many researchers have addressed this challenge through the application of convolutional neural networks (CNNs) on RGBD images. Refs. [18,19] incorporate both RGB and depth information at various stages. These methods initially predict the coarse 6D pose of the object based on the RGB image and subsequently employ the ICP algorithm with depth information to optimize the pose of the target object. They will result in more accurate pose estimation than traditional methods. However, these methods suffer from time-consuming refinement phases, preventing them from achieving real-time inference speeds [20,21]. Recently, DenseFusion [22] integrated an end-to-end iterative pose refinement process to enable real-time inference through a better fusion strategy. As shown in Figure 1a, DenseFusion extracts appearance and geometry information independently from RGBD images via a separate network. These features are then fused at the pixel level to estimate the 6D object pose. However, in the final fusion of dense features, the two modes are simply concatenated. The concatenated features may lack discriminative power, leading to inaccurate pose estimation between objects with similar appearances. This is because the separation of CNN-based feature extraction and point cloud-based feature extraction may not adequately capture the complex and complementary information from both modalities. Therefore, as shown in Figure 1b, our approach focuses on capturing information within and between modalities. Moreover, due to the quality problem of the obtained RGBD image and the complexity of the experimental environment, the contribution degree of each modality differs in the target task. Treating the contribution of each modality as the same will lead to a large error in the task of estimating the 6D pose of target objects under adverse conditions.

In this work, we propose a new end-to-end network based on 6D pose estimation using RGBD images. The network models the correlation between RGB and depth features, then the attention mechanism is used to help select salient features present in both modalities to improve the 6D pose estimation performance. The core of our method considers the information interactions within and between modalities and obtains the contribution degrees of different modalities to the target task using CWTM. We use encoders to extract features from RGB and depth images separately, adding the efficient channel attention module (ECAM) and sampling center self-attention module (CSAM) to efficiently extract modality internal information. Thus, we can obtain pixel-level multimodal features that preserve the appearance and geometric information. One modality is regarded as the primary input and the other modality is regarded as the secondary input in CFFM; thus, we can obtain the interaction information between modalities. After concatenating the features of the two modalities with interaction information, CWTM is used to obtain features with different weights. The features of individual pixel points are fed into the pose estimation network for pose prediction.

In summary, our method can efficiently fuse RGB and depth features on the extracted pixel points of the target object. Thus, our method resists heavy occlusion and achieves accurate 6D pose estimation without additional refinement. The contributions of our work can be summarized as follows:

- A new cross-modality feature fusion method is proposed. Firstly, ECAM and CSAM are added to the feature extraction network to obtain contextual information within a single modality; secondly, CFFM is set to obtain interaction information between RGB and depth modalities so that cross-modality features can be extracted and fused efficiently at the pixel level to improve the accuracy of 6D object pose estimation;
- CWTM is proposed to obtain the contribution degrees of different modality data of 6D pose estimation tasks for target objects. The contextual information within each pixel neighborhood is fully utilized to achieve an accurate pose without additional refinement processes;
- Our method is evaluated on the LineMOD dataset [10] and YCB-Video dataset [23]. Better results are demonstrated on smoothed and textureless objects in the YCB-Video dataset and the LineMOD dataset.

## 2. Related Work

### 2.1. Pose Estimation Based on RGB Data

The classical LineMOD method [24] utilizes the gradient angle method to quantify and match the similarity with the template, ultimately obtaining the pose estimate of the object. While this method is fast, it does not perform well on low-texture objects. In current research, deep learning techniques are widely employed to extract object features using CNNs and directly estimate object pose. For instance, SSD-6D [25] utilizes different convolution kernels to obtain multi-scale features and employs a sliding window approach to generate classification and regression results for each pixel on the feature map. The method exhibits better detection performance on objects with rich color information. PoseCNN [23] is a method that estimates the 6D pose of an object using a single RGB image and the pose is optimized via the ICP algorithm. To handle symmetric objects, PoseCNN introduces a new loss function, which is also used in our paper. PVNet [26] is a method that performs regression on the pixel unit vectors of key points. Using these vectors, the random sample consensus (RANSAC) algorithm is applied to directly determine the key point’s location, which enhances resistance to occlusion and truncation. Ref. [27] improves the key point prediction accuracy and adds transformers for location coding. Ref. [28] incorporates a segmentation attention module; moreover, a multi-level information fusion module removes redundancy. However, due to the absence of depth information, the detection effectiveness is inferior to methods based on RGBD data.

### 2.2. Pose Estimation Based on Point Clouds

With the advancements made in point cloud technology [29,30], researchers [31,32] have explored converting depth maps into point clouds and extracting features or 3D bounding boxes from these point clouds in 6D object pose estimation. A recent method [33] utilized depth images for class-level 6D object attitude estimation. It leverages point cloud geometry to capture instance consistency and differences, resulting in accurate predictions for the positioning and orientation of unseen object instances. However, one limitation of this approach is that depth sensors cannot effectively capture objects with reflective surfaces. This limitation emphasizes the need to consider RGB images in object pose estimation.

### 2.3. Pose Estimation Based on RGBD Data

The emergence of depth sensors, like RealSense and Kinect, has enabled deep learning models to effectively utilize RGBD information for pose estimation. In [34], the colors and depths of these patches are normalized and regressed using a voting algorithm that can extend to multiple object classes. However, the depth information is considered an additional channel to the RGB channel. To achieve comprehensive integration of cross-modality information, ref. [18] employed a multi-stage approach that combined RGB and depth information. However, it is important to note that these methods have undergone a time-consuming refinement process. To solve this, ref. [22], respectively, processed RGB images and point clouds independently via CNN and PointNet architectures. To select the best prediction results, PointFusion [35] uses input 3D points as spatial anchors to predict multiple 3D box hypotheses and their confidence levels after processing the RGB and depth information separately. PVN3d [36] uses a deep network to detect the 3D key points of an object from the RGBD image and then estimates the 6D pose using a least-squares fitting manner. These methods use an end-to-end iterative pose optimization process to enable real-time inference. However, one limitation of these methods is that features do not fully interact when extracting and fusing features. As a result, the expressive power of learning representations is limited.

### 2.4. Summary of 6D Object Pose Estimation Methods

Table 1 classifies the above methods according to the input and research methods. Our approach is based on RGBD data and deep learning.

## 3. Method

### 3.1. Overview of Our 6D Pose Estimation Method

The goal of our network is to estimate the 6D pose of a target object from an RGBD image. Specifically, the 6D pose refers to a homogeneous transformation matrix P=[R|t], which is composed of a translation transformation $t\in R^{3}$ and a rotation transformation R∈SO(3). Since a 6D object pose is estimated from a camera image, it involves a transformation of the 3D object coordinate system to the camera coordinate system.

**Figure 2:**
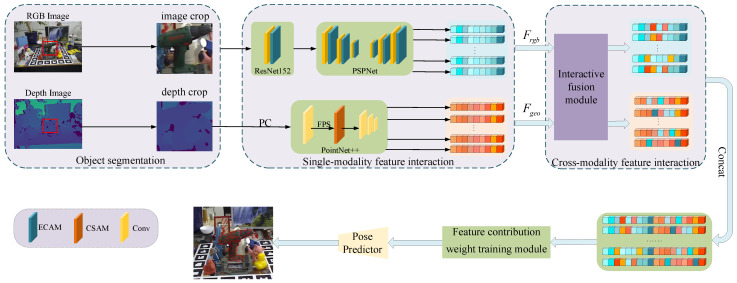
Overview of our 6D pose estimation method.

Figure 2 shows the overall structure of the end-to-end network estimating the 6D pose, and it is divided into five main stages. In the first stage, the input RGB and depth image are semantically segmented, so as to obtain the RGB and depth image corresponding to the target object. The second stage sends the results of the first stage to different convolutional networks to extract features, respectively. In the feature extraction process, the depth image is converted into a point cloud to obtain geometric information. We consider the contextual information between features within a single modality. In the third stage, the features outputted in the second stage are fed to CFFM to obtain the association information between cross-modal modalities, enabling better fusion of the two modalities at the pixel level. In the fourth stage, the CWTM is used to obtain the degree of contribution of each modality of the task. The degree of contribution involves the importance of the distribution probability. The distribution probabilities are obtained by taking the dot product with multimodal features. After reweighting the importance of different modality features, we obtain the new cross-modality fusion features. Finally, the cross-modality fusion features are fed into the pose prediction network to estimate the 6D object pose, and the result with the highest confidence is output.

### 3.2. Semantic Segmentation Module

In the first stage, we employ an aligned RGBD image obtained from a depth camera as input. Firstly, we adopt semantic segmentation on the RGB image to extract the target region. To balance efficiency and performance, we directly utilize an existing semantic segmentation network [23]. Next, based on the object bounding box derived from the RGBD image segmentation, we crop the RGB and depth images separately. In our approach, the cropped RGB and depth images serve as inputs to the network for extracting appearance and geometric features of the target object. This approach combines both appearance and geometric information, providing valuable input for the subsequent estimation of the 6D object pose.

### 3.3. Feature Extraction Module

In the second stage, we transform the depth information into point cloud information. The cropped depth image is converted into a 3D point cloud by the camera’s internal reference matrix. The input point cloud $P=\{p_{i}={\left[X_{i},Y_{i},Z_{i}\right]}^{T}\in R^{3}|i=1,\cdots ,N\}$ contains N points, representing the 3D spatial coordinates of the th point in the input point cloud. The input RGB image is an image of size H×W×3 obtained by cropping the enclosing frame of the semantic segmentation mask. Although the depth information provides additional geometric information to help solve the problem, the RGB and depth information exist in two different spaces. Therefore, how to effectively fuse the intermodal features across modalities is the key to an accurate 6D pose estimation.

We obtain features with contextual information within a single modality. Since the two information representations are different, we use CNN to extract appearance features of the RGB image and PointNet++ network to extract the geometric features of the input point cloud.

#### 3.3.1. RGB Image Feature Extraction Network

The image feature extraction network structure consisting of ResNet152 [37] and PSPNet [38] is referred to as the image feature extraction method in [22]. The structure is shown in the image feature extraction module in Figure 2. The ResNet152 network preprocesses the RGB image, and the PSPNet network is used for multi-scale feature extraction.

To obtain the contextual information within the feature channels, we add ECAM to each bottleneck of the ResNet152 network. The commonly used channel attention mechanism is SeNet [39], whose key operations are squeeze and escape. However, it was found through research [40] that the dimensionality reduction operation affects the channel attention performance, while proper cross-channel interactions can greatly reduce the complexity of the model and maintain efficient performance. Therefore, in our method, the strategy involving no dimensionality reduction is used in the basic attentional structure.

**Figure 3:**
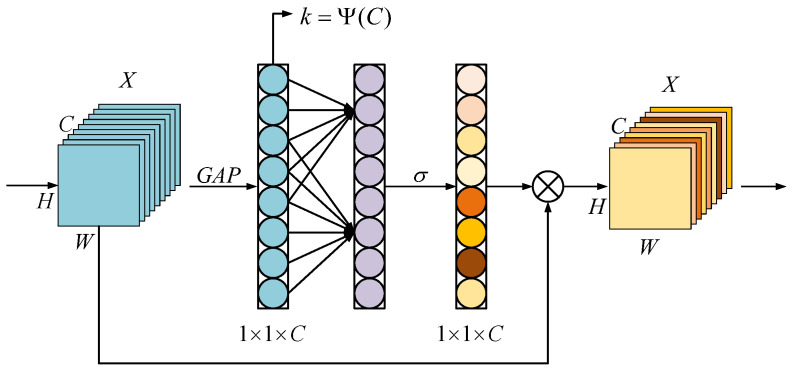
Efficient channel attention module (ECAM).

As shown in Figure 3, the input of ECAM is *X* with a channel dimension of C and a size of H × W, which is the output of the last convolution block. Firstly, features are extracted by convolutional kernels of different sizes to capture local cross-channel interaction information. Therefore, the size of the convolutional kernel *k* represents the coverage of cross-channel interactions. And *k* is the number of neighbors involved in channel-specific attention prediction. Therefore, the interaction coverage needs to be determined. These researchers [41,42] use group convolution to improve the CNN architecture, where a fixed number of long-range (short-range) convolution groups for high-dimensional (low-dimensional) channels are shown to yield good results. Similarly, *k* is proportional to the channel dimension *C*. This suggests the existence of a mapping ϕ between *k* and *C*, which is expressed as follows:

(1) C=ϕ(k)

The exponential function in efficient channel attention is used to approximate the mapping, as shown in Equation (2). In addition, since the channel dimension is usually set to the integer power of 2, the mapping relation of Equation (3) is obtained by replacing exp(γ×k−b) with $2^{\left(\gamma \times k-b\right)}$.

(2) C=ϕ(k)≈exp(γ×k−b)

(3) $$C=\phi \left(k\right)=2^{\left(\gamma \times k-b\right)}$$

Then, given the channel dimension, the kernel size can be adaptively determined as

(4) $$k=\Psi \left(C\right)={\left|\frac{\log_{2}\left(C\right)}{\gamma }+\frac{b}{\gamma }\right|}_{odd}$$

where ${\left|m\right|}_{odd}$ represents the odd number closest to m. We, respectively, set γ and *b* to 2 and 1 in all experiments. Clearly, by mapping the nonlinear mapping Ψ, the high-dimensional channels have longer distance interactions, while the low-dimensional channels have shorter distance interactions.

Secondly, the attention vector is normalized using the sigmoid activation function, ensuring that the attention weights for each channel range from 0 to 1. Each channel of *X* is multiplied by the corresponding attention weight, achieving channel-wise feature weighting. Finally, the weighted *X* is used as the output of the model for further processing by subsequent layers.

#### 3.3.2. Point Cloud Feature Extraction Network

After converting the depth image into point clouds, we use the PointNet++ network to extract the point cloud features. The PointNet++ network uses farthest point sampling (FPS) to divide the point cloud data into different sub-regions. However, due to the differences in point cloud densities in different regions and the sparseness of the sampled point cloud data, the adjacency relation in the original point cloud data is corrupted to some extent. This affects the representation of the target’s geometric feature structure. In order to solve this problem and obtain the contextual information inside the point cloud, we introduce sampling center self-attention. The sampling center self-attention module (CSAM) is concerned with the geometric relationship between sampling center points and their neighbors, and the key lies in how to reasonably encode the spatial relationships in sampling point neighborhood topology. The point cloud $U=\{f_{i}|i=1,\cdots ,n\}$ is obtained by FPS in the PointNet++ network, where *n* is the number of points, $f_{i}$ denotes the combination of spatial coordinates and attribute features of the *i*th point, $f_{i}=\left[p_{i},q_{i}\right]\in R^{3+d}$, $p_{i}=\left(x_{i},y_{i},z_{i}\right)\in R^{3}$ denotes the spatial coordinates of the *i*th point, and $q_{i}\in R^{d}$ denotes the J-dimensional attribute features stored in the *i*th point cloud. Then, the *K* nearest neighbor (KNN) algorithm is used to obtain the *K* nearest neighbors of the central sampling point, and it completes the single-scale local neighborhood division of the input point set. Inspired by RandLA-Net [43], we specify the geometric relationship between the sampled centroids and their neighbors as the Euclidean measure, the direction vector, and the spatial location of each point. Thus, the feature vector is spatially encoded as

(5) $$\mu_{i}^{k}=\mathrm{MLP}\left(p_{i}⊕p_{i}^{k}⊕\sqrt{{\left(p_{i}-p_{i}^{k}\right)}^{2}}⊕\left(p_{i}-p_{i}^{k}\right)\right)\;$$

where k∈[0,K], MLP represents multilayer perceptron, $p_{i}\left(i\in n\right)$ and $p_{i}^{k}$, respectively, represent the spatial coordinates of the center point and the neighboring points, ⊕ denotes the concatenated feature, $\sqrt{{\left(p_{i}-p_{i}^{k}\right)}^{2}}$ denotes the Euclidean metric between the center sampling point and the neighboring point, and $\left(p_{i}-p_{i}^{k}\right)$ denotes the relative displacement direction vector. After the above equation, ten-dimensional features are selected for each neighborhood point as the result of local spatial position encoding.

Next, we calculate the self-attention score of the feature vector and aggregate it to obtain the output of the central self-attention mechanism that reflects the significance of the geometric structure of the neighborhood of sampled points. The calculation procedure is as follows:

(6) $$Q=g\left(\mu_{i}^{k}\right)$$

(7) $$K=u\left(\mu_{i}^{k}\right)$$

(8) $$V=v\left(\mu_{i}^{k}\right)$$

(9) $$A=K^{T}Q$$

(10) $$A^{{}^{\prime }}=\mathrm{softmax}\left(\frac{A}{\sqrt{C}}\right)$$

(11) $$\hat{f}=A^{{}^{\prime }}\cdot V$$

where $g\left(\cdot \right)$, $u\left(\cdot \right)$, and $v\left(\cdot \right)$ are the 1×1 convolution operations; they convert $\mu_{i}^{k}$ into *Q*, *K*, and *V* in the self-attention mechanism. Although *Q*, *K*, and *V* are all obtained from $\mu_{i}^{k}$ by the 1×1 convolution operation, the three are not the same. *A* denotes the similarity between features after the convolution of $\mu_{i}^{k}$, *C* denotes the number of output channels, *A* divides $\sqrt{C}$, playing a moderating role in making the gradient more stable during training, softmax is an activation function, $A^{{}^{\prime }}$ is the self-attentive score of feature vector $\mu_{i}^{k}$, and $\hat{f}$ is the output feature of the central self-attentive mechanism.

**Figure 4:**
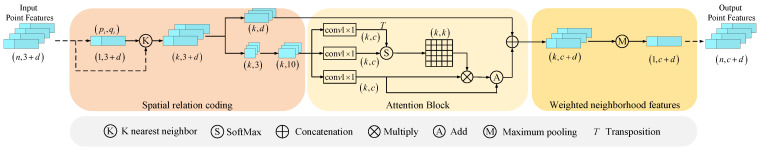
Sampling center self-attention module (CSAM).

As shown in Figure 4, the dimensionality of the original point cloud feature input is 3+d, where *N* is the number of point clouds, 3 represents the dimensionality of the spatial coordinates of the point cloud features, and *d* represents the dimensionality of the attribute features of the point cloud. In the spatial encoding module, we first input the *i*th point into the KNN sampling network to aggregate its *K* neighboring points, then separate the spatial coordinate features from the attribute features. The spatial coordinate features are re-coded into ten dimensions, and we aggregate the spatial feature vectors in the self-attentive module. The resulting output is concatenated with the attribute features. The aggregated features are weighted by the maximum pooling of all data features within the neighborhood of the centroid to obtain the global features in that neighborhood. Finally, the weighted neighborhood features of each centroid are output as the output point cloud features.

### 3.4. Cross-Modality Feature Fusion Module

**Figure 5:**
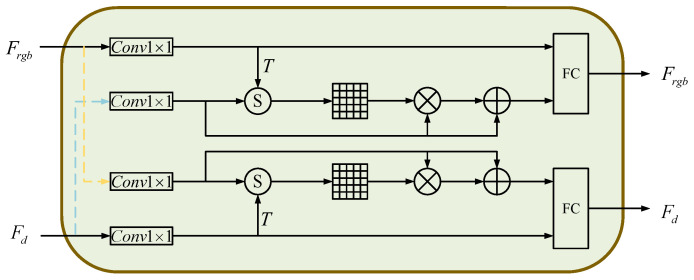
Cross-modality feature fusion module (CFFM).

Since the number of features extracted in the point cloud and RGB image are different, we fuse the selected point clouds with their corresponding color features at the same location. In our method, the cross-modality feature fusion module (CFFM) is designed to fuse cross-modality features. The details of CFFM are shown in Figure 5. We use the features obtained from the two feature extraction modules as the input to the feature fusion module. Meanwhile, one of the features is used as the primary input and the other as the auxiliary input; the two input modalities are fused to generate the target modal output. Let the primary input be $E=\left\{E_{1},E_{2},\ldots ,E_{n}\right\}\in R^{d_{e}\times n}$ and the auxiliary output be $G=\left\{G_{1},G_{2},\ldots ,G_{n}\right\}\in R^{d_{t}\times n}$. We project the primary input *E* and the auxiliary input *G* into the same shared vector space:

(12) $$E_{emb_{i}}=\tanh\left(P_{E_{emb}}E_{i}+C_{E_{emb}}\right)$$

(13) $$G_{emb_{i}}=\tanh\left(P_{G_{emb}}G_{i}+C_{G_{emb}}\right)$$

where i∈[1,n], tanh is an activation function. $P_{E_{emb}}\in R^{d_{v}\times d_{e}}$, $P_{G_{emb}}\in R^{d_{v}\times d_{l}}$, $C_{E_{emb}}\in R^{d_{v}}$, $C_{G_{emb}}\in R^{d_{v}}$ are the training parameters and $d_{v}$ denotes the dimensionality of the shared vector space. We use $E_{emb}$ and $G_{emb}$ to calculate the attention matrix $M\in R^{n\times n}$, $M_{ij}$ denotes the correlation between the *i*th content of the primary input and the *j*th content of the secondary input, and the attention matrix *M* can be expressed as

(14) $$M_{ij}={E_{emb_{i}}}^{T}\cdot G_{emb_{i}}$$

To measure the importance of each auxiliary input to the primary input, we use the softmax function to quantify *M*:

(15) $$M_{ij}=\frac{\exp(M_{ij})}{\sum_{j=1}^{n}\exp(M_{ij})}$$

Then we can obtain the auxiliary input based on the attention mechanism *J*:

(16) $$J=G\cdot M^{T}$$

Finally, the primary input *E* and the secondary input *J* are spliced in the fully connected layer to obtain the fused feature $F=\left\{F_{1},F_{2},\ldots ,F_{n}\right\}$:

(17) $$F=\tanh\left(P_{f}\left[E_{i}:J_{i}\right]+C_{f}\right)$$

where $P_{f}$ and $C_{f}$ are the training parameters. When the main input *E* is $F_{rgb}$, $P_{f}\in R^{d_{e}\times \left(d_{e}+d_{t}\right)}$, $C_{f}\in R^{d_{e}}$; when the main input is $F_{d}$, $P_{f}\in R^{d_{t}\times \left(d_{e}+d_{t}\right)}$, $C_{f}\in R^{d_{t}}$.

By using CFFM, we obtain the fused features. They are partly point cloud-dominated–RGB image-supplemented features and partly RGB image-dominated–point cloud supplemented features. The two fused features are input to the fully connected layer and connected to obtain the output features after the interaction between the two modalities.

### 3.5. Feature Contribution Weight Training Module

The two modalities interact for single-modality and cross-modality information. But for the task of 6D object pose estimation, the feature contribution degree is different for each modality. Considering this problem, we use the feature contribution weight training module (CWTM) to optimize the weights of the features through network adaptive generation. That is to say, CWTM automatically selects the most appropriate modal features from the modalities, allowing the model to be robust and adaptive.

**Figure 6:**
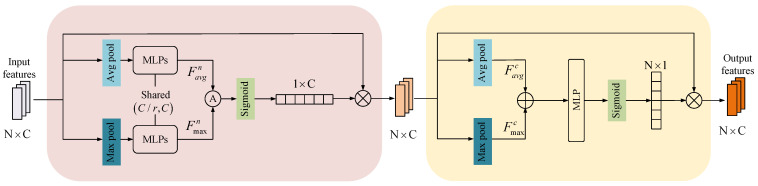
Feature contribution weight training module (CWTM).

As shown in Figure 2, we form a multimodal joint feature $F\in R^{n\times C}$ after a concatenate operation of the interacted modal features, where *n* denotes the number of features and *C* denotes the number of feature channels. In Figure 6, when pooling the number of feature *n* dimensions, the features can be expressed as follows:

(18) $$F_{n}=M_{n}\left(F\right)⊗F$$

where $M_{n}\in R^{1\times C}$ denotes the feature channel attention mechanism, $F_{n}$ denotes the output features of channel attention, and ⊗ denotes matrix fork multiplication.

To generate $M_{n}$, with the experience of CBAM, the use of different pooling methods implies that the feature information is collected through different perspectives. Different pooling can effectively improve the network’s expressiveness. We perform feature aggregation along the number of feature *n* dimensions using parallel average pooling and maximum pooling on the input features *F* to generate feature representations from different angles, $F_{avg}^{n}$ and $F_{\max}^{n}$. The aggregated features are trained using a double-hidden-layer MLP with shared parameters. Then the feature channel *C* is scaled and recovered, and the scaling factor is *r*. Moreover, *r* is used to generate attention weights. Finally, the sigmoid function is used to activate. The feature channel attention mechanism *M* can be expressed as follows:

(19) $$M_{n}=\sigma \left(MLP(AvgPool(F))+MLP(MaxPool(F))\right)=\sigma \left(W_{1}\left(W_{0}\left(F_{avg}^{n}\right)\right)+W_{1}\left(W_{0}\left(F_{\max}^{n}\right)\right)\right)$$

where AvgPool denotes the average pooling, MaxPool denotes max pooling. σ denotes the sigmoid activation function, $W_{0}$ and $W_{1}$ denote the weights of the MLP, $F_{avg}^{n}$ and $F_{\max}^{n}$ denote the average pooling and maximum pooling operations for feature *F* along the number of feature *n* dimensions.

The specific process is shown in Figure 6. Next, to obtain the spatial attention of features, we use $F_{n}$ along the feature channel *C* for feature aggregation, using parallel average pooling and maximum pooling to generate feature representations $F_{avg}^{c}$ and $F_{\max}^{c}$ from different angles. The two aggregated features are subjected to the operation of summation and their dimensions are adjusted by the MLP network; finally, they are input to the sigmoid function to obtain the spatial attention mechanism $M_{c}$. $F_{n}$ denotes the output of the spatial attention of features. The calculation formula is as follows:

(20) $$\begin{array}{c} F_{c}=M_{c}\left(F_{n}\right)⊗F_{n} \end{array}$$

(21) $$\begin{array}{c} M_{c}=\sigma \left(MLP(AvgPool\left(F_{n}\right)+MaxPool\left(F_{n}\right))\right)=\sigma \left(W\left(F_{avg}^{c}+F_{\max}^{c}\right)\right) \end{array}$$

where *W* denotes the weight of the MLP.

### 3.6. Object 6D Pose Estimation

The 6D pose estimation network consists of four fully connected layers. It directly outputs the pose prediction value with a confidence level for each pixel point’s fused feature. We will select the pose predicted by the pixel with the highest confidence level as the predicted pose of the object.

After establishing the network architecture, the next step is to define the loss function. In our method, the primary goal is to estimate the 6D pose of the object. To achieve this, we define the pose estimation loss as the distance between the sampled points of the real pose in the object model and the corresponding points transformed from the predicted pose to the same model. Consequently, we define the per-pixel predicted minimization loss as follows:

(22) $$\begin{array}{c} L_{i}^{p}=\frac{1}{n}\sum_{j}||(Rx_{j}+t)-\left(\hat{R_{i}}x_{j}+\hat{t_{i}}\right)\left|\right| \end{array}$$

where $x_{j}$ represents the *j*th point sampled in the target model, p=[R|t] and $\hat{p_{i}}=\left[\hat{R_{i}}|{\hat{t}}_{i}\right]$ each represent the ground pose and predicted pose of the *i*th feature point, and *n* represents the number of sampled points in the target model.

For symmetric objects, Equation (22) assumes that a single correct rotation may not be suitable due to the presence of multiple valid rotations. To address this, we define a loss function that incorporates symmetry estimation to better handle symmetric objects and achieve more accurate pose estimations:

(23) $$\begin{array}{c} L_{i}^{p}=\frac{1}{n}\sum_{j}\min_{0<k<n}\left|\right|\left(Rx_{j}+t\right)-\left(\hat{R_{i}}x_{k}+\hat{t_{i}}\right)\left|\right| \end{array}$$

To improve the pose estimation, we incorporate pixel point confidence into the loss function of the network. This introduces weights to each pixel loss and includes a confidence regularization term, enhancing the accuracy and robustness of the estimation. Consequently, the final loss function is:

(24) $$\begin{array}{c} L=\frac{1}{N}\sum_{i}\left(L_{i}^{P}c_{i}-\omega \log\left(c_{i}\right)\right) \end{array}$$

where $c_{i}$ denotes the confidence level of the *i*th feature point and ω denotes the balanced hyperparameter. At low confidence levels, the penalty term ω will be larger. We use the estimated result with the highest confidence level as the final output.

## 4. Experiments

### 4.1. Datasets

We evaluate our method on two benchmark datasets.

LineMOD is a dataset consisting of 13 low-texture objects from 13 videos, with a total of 15,783 frames. The lack of textured objects, cluttered scenes, and variable lighting make this dataset challenging. We use the same training and testing partitions as in previous work [22], where 85% of the dataset is used for training and the remaining 15% for testing.

The YCB-Video dataset is a more challenging dataset that contains 21 YCB objects of different shapes and textures. It consists of 92 RGBD videos, of which, 16,189 frames from 80 videos are selected for training with 80,000 synthetic frames; 2949 keyframes from the remaining 12 videos are selected for testing.

### 4.2. Evaluation Metrics

We use the ADD and ADD-S metrics for evaluation. For non-symmetric objects, the ADD metric measures the average distance between the point pairs of object vertices transformed by the predicted and ground truth poses, defined as follows:

(25) $$\begin{array}{c} \mathrm{ADD}=\frac{1}{m}\sum_{\upsilon \in o}∥\left(R\upsilon +T\right)-\left(R^{*}\upsilon +T^{*}\right)∥ \end{array}$$

where m represents the number of points in the point cloud *o*; υ is the point in object *o*; *R* and *T* are the predicted pose transformation matrices; and $R^{*}$ and $T^{*}$ are the true pose transformation matrices.

For symmetric objects, the ADD-S metric based on the nearest point distance is used to evaluate:

(26) $$\begin{array}{c} \mathrm{ADD}\text{-}S=\frac{1}{m}\sum_{\upsilon_{1}\in o}\min_{\upsilon_{2}\in o}∥\left(R\upsilon_{1}+T\right)-\left(R^{*}\upsilon_{2}+T^{*}\right)∥ \end{array}$$

In the YCB-Video dataset, ADD-S calculates the mean distance from each 3D model point transformed by [R|t] to its closest neighbor on the target model transformed by $\left[R^{*}|t^{*}\right]$. ADD-S takes symmetric and non-symmetric objects into the overall evaluation. We obtain the area under the ADD-S curve (AUC) following PoseCNN. We also report on the percentage of ADD-S that is less than 2 cm (<2 cm), which measures the number of predicted grasping successes of the robot at a minimum tolerance.

In the LineMOD dataset, we use ADD for non-symmetric objects and ADD-S for symmetric objects. We specify a distance accuracy of less than 10% of the object diameter.

### 4.3. Experimental Details

We implemented our method in the PyTorch framework and trained it on an Ubuntu 18.04 system and an NVIDIA GeForce RTX 3090Ti GPU. In the training process, we used Adam with an initial learning rate of 0.0001 and a batch size of 8 to train the network.

The maximum number of iterations was set to 500, the batch size was set to 16, the hyperparameter was set to 0.015, and the number of pixel features was set to 500.

### 4.4. Evaluation of the LineMOD Dataset

On the LineMOD dataset, the pose estimation is considered correct when ADD (-S) is less than 10% of the object’s diameter, and the ADD (-S) score is defined as the percentage of correct pose estimations. Here, ADD (-S) denotes the ADD and ADD-S metrics for both non-symmetric and symmetric objects.

The results in Table 2 show that the average score of our method is 96.9%. When comparing our method using all modules with PoseCNN+ICP, PVNet, SSD6D+ICP, PointFusion, and DenseFusion, it is 8.3%, 13.6%, 20.2%, 23.2%, and 2.6% higher, respectively. In the methods using an RGB image as the input, PVNet and PoseCNN+ICP perform well. However, due to the lack of spatial information, their performances are poorer than DenseFusion using an RGBD image as input. In a 2D projection with 3D key points predefined by the SSD6D+ICP network using an RGBD image as input, it is impossible to avoid the result of the projection error. PointFusion performs better in large scenes but is less accurate in estimating the pose of an individual object. The average score of the method used in our method improves by 2.6% compared to DenseFusion and significantly outperforms other methods. In particular, the performance improvement is significant for low-texture objects, such as drill bits (7.8%), spray cans (4.0%), and cell phones (4.4%). It is shown that the method can extract and fuse multimodal features on a pixel-by-pixel basis more effectively.

**Figure 7:**
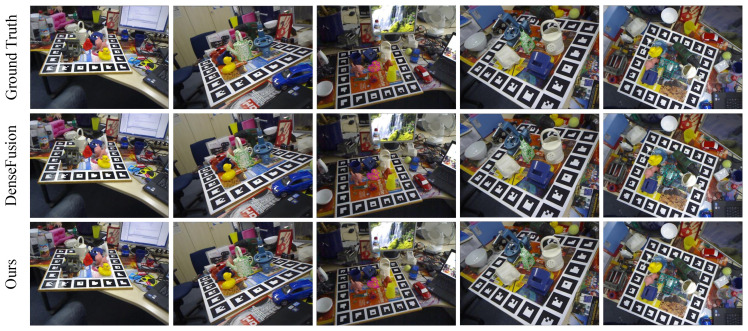
LineMOD dataset visualization results.

The visualization of the LineMOD dataset results is shown in Figure 7. We rotate and translate the points in the selected point cloud model and project them onto the RGB image. Figure 7 shows some sample estimates (ape, can, duck, glue, cam) produced by DenseFusion and our method. The pose estimation results of our method on ‘ape’ and ‘can’ are much closer to the true values. The point cloud model projection basically overlaps with the target object in the image, and it is significantly better than the DenseFusion method.

**Figure 8:**
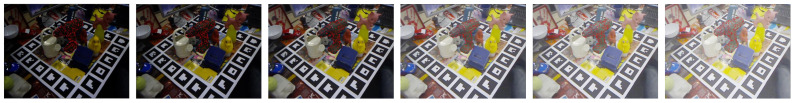
Pose estimation results of the driller under different lighting conditions.

The RGBD camera captures images in dark or bright lighting conditions, which only affects the RGB image and not the depth image. Therefore, we verify the robustness of our method under illumination conditions by varying the luminance value of the RGB image in the same scene. Figure 8 shows the pose estimation results of our method for the driller under changing illumination conditions. It can be seen that our method incorporates the RGB and depth image features, and can solve the pose estimation problem under the changing illumination conditions.

### 4.5. Evaluation of the YCB-Video Dataset

The quantitative evaluation results of ADD-S AUC and ADD-S scores (<2 cm) for all 21 objects on the YCB-Video dataset are presented in Table 3. To ensure the fairness of the comparison, we used the same semantic segmentation mask of PoseCNN. As shown in Table 3, our method outperforms the state-of-the-art method on most of the objects. Moreover, in terms of average accuracy, it outperforms PoseCNN+ICP by 1.7% and 3.7% in two metrics, DenseFusion by 3.5% and 1.6%, and YOLOPose V2 by 4.6% on the ADD-S AUC metric. Since there are objects in the YCB-Video dataset with different illumination and occlusion conditions, this shows that our method is more robust to illumination and occlusion variations. The bowl in the YCB-Video dataset belongs to the smooth and textureless object. For such objects, our method is able to improve the accuracy of pose estimation, indicating that the performance of the network can be improved by fusing the appearance and geometric features of cross-modalities.

**Figure 9:**
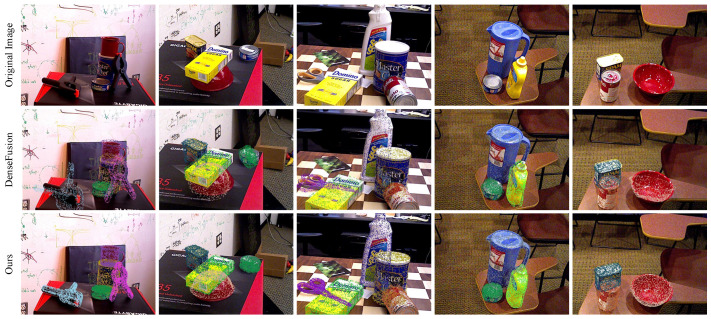
Visualization results of the YCB-Video dataset.

We also show the qualitative analysis of the YCB-Video dataset by rotating and translating the points in the selected point cloud model and projecting them onto the corresponding RGB image to obtain the visualization of the pose estimation results. The visualization results of our method on the YCB-Video dataset are shown in Figure 9 and are compared with the DenseFusion method. The visualization results show that our method performs well on asymmetric objects, and the point cloud model projection mostly overlaps with the corresponding image, while DenseFusion cannot accurately estimate objects, such as large_clamp, extra_large_clamp, and ‘bowl’ due to occlusion, etc.

### 4.6. Noise Experiment

In a real-world operating environment, the presence of sensor noise can seriously affect the quality of depth images captured by the RGBD camera. Therefore, during network training, we add random noise to the depth images. As noise is added, the robustness of our method to noise increases. As shown in Table 4, when the depth image is affected by random noise in the range of (−20.0, 20.0), the pose estimation results can still be estimated accurately.

### 4.7. Ablation Experiments

Compared with the most relevant DenseFusion method, our method has three improvements. Firstly, we consider the information within a single modality to efficiently extract RGB image features and point cloud features; secondly, we present CFFM, which uses the interaction information from cross-modalities to efficiently extract and fuse multimodal features pixel-by-pixel; finally, we propose CWTM, which computes the different contributions of features to the target task.

To illustrate the impacts of the three modules, we, respectively, conducted ablation methods on the LineMOD and YCB-Video datasets. The results are presented in Table 2 and Table 3. As shown in Table 2, the average ADD(-S) score of the method using only the feature extraction module is 93.2%. This value increases to 95.3% when CFFM is added, at which point, it outperforms DenseFusion by 1%, PoseCNN+ICP by 6.7%, PVNet by 9%, SSD6D+ICP by 18.6%, and PointFusion by 21.6%. After CWTM is added, the average score of our method is 96.9%. As shown in Table 3, using feature interaction in the single modality, the ADD-S score (<2 cm) of our method is 0.4% higher than that of YOLOPose V2. Using only the feature extraction module, the ADD-S score (<2 cm) of our method is 0.4% higher than that of PoseCNN+ICP. The performance is improved with the addition of the cross-modality feature fusion module. We believe that our approach adequately fuses the appearance and geometric features to help the model identify which part of the object the point belongs to. When all three modules are used, the average ADD-S AUC and ADD-S scores (<2 cm) of our method are 1.7% and 3.3% higher than PoseCNN+ICP, and 3.5% and 1.2% higher than DenseFusion.

Our experiments demonstrate that our cross-modality feature fusion network effectively integrates RGB and depth information. Furthermore, our network exhibits commendable performance even in scenarios where the objects are occluded or in low-light environments.

### 4.8. Robotic Grasping Experiment

In order to assess the accuracy of the grasping posture estimated by our method, we conducted experiments in real grasping scenarios. The algorithm proposed in this paper was deployed on the TA6R3RevB1 six-axis robot arm system. We utilized the D435i camera as the vision sensor, employed a three-finger flexible clamp as the end actuator, and controlled the robot arm using the robot operating system (ROS) to grasp the target objects. For the experiment, we selected five objects from the YCB-Video dataset. These objects were placed on a table in random orientations and there was mutual obstruction between the objects. The robotic arm attempted to grasp each object 12 times, resulting in a total of 60 grasp attempts. Notably, we achieved a commendable success rate, with the robotic arm successfully grasping the target objects in 81% of the attempts. It is worth mentioning that the choice of the claw significantly influenced the success rate of grasping. In some cases, the claw’s movement inadvertently caused the object to shift, leading to grasp failure. However, our method accurately predicted the attitudes of the target objects despite these challenges, demonstrating the robustness and effectiveness of our approach.

### 4.9. Limitations

Our network is currently limited by two factors. Firstly, the network was trained on the YCB-Video dataset using the segmentation masks provided by PoseCNN [23]. Since PoseCNN primarily focuses on semantic segmentation, the segmentation quality for instance objects is not optimal. Consequently, the performance of our network may decrease when dealing with large_clamp and extra_large_clamp objects. Secondly, our network encounters challenges when handling symmetric objects due to the inherent ambiguity in their orientation. This ambiguity leads to less accurate pose prediction compared to objects with clear non-symmetry. For instance, when dealing with symmetric objects like the bowl, although our results outperform other methods, they are not as accurate as those achieved with non-symmetrical objects.

## 5. Conclusions

In this paper, we propose a novel 6D object pose estimation method from a single RGBD image. Our method involves extracting and aggregating contextual information within a single modality and cross-modality interactive information at the pixel level. The cross-modality feature fusion method ensures the comprehensive fusion of both RGB and depth modalities. Furthermore, different modality contributions are considered to achieve accurate pose estimation. The experimental results show that our method excels in performance on both the LineMOD dataset and the more challenging YCB-Video dataset, surpassing the majority of existing pose estimation methods. In future work, our main focus will be to improve segmentation for instance objects and solve the ambiguity problem in symmetric target direction estimation.

## Figures and Tables

**Table 1:** Summary of 6D object pose estimation methods described above.

Methods	Traditional Methods	Deep Learning-Based Methods
RGB data-based methods	LineMOD [24]	SSD-6D [25], PoseCNN [23], PVNet [26], YOLOPose V2 [27], SANet [28]
RGBD data-based methods	[1,14,15]	DenseFusion [22], PointFusion [35], PVN3d [36], our method

**Table 2:** Quantitative evaluation of 6D pose based on the ADD (-S) metric on the LineMOD dataset. The objects with names in bold font indicate symmetry. ‘Inter’ indicates the modality intra-feature interaction module, ‘cross’ indicates the cross-modality feature interaction module, and ‘weight’ indicates the attentional weight training module.

	PoseCNN + ICP	PVNet	SSD6D + ICP	Point Fusion	Dense Fusion	Ours (Inter)	Ours (Inter + Cross)	Ours (Inter + Cross + Weight)
ape	77.0	43.6	65.0	70.4	92.3	88.2	89.1	95.8
ben.	97.5	99.9	80.0	80.7	93.2	91.5	93.5	94.1
cam.	93.5	86.9	78.0	60.8	94.4	93.4	96.3	97.6
can	96.5	95.5	86.0	61.1	93.1	93.8	97.0	97.7
cat	82.1	79.3	70.0	79.1	96.5	94.4	96.4	97.6
driller	95.0	96.4	73.0	47.3	87.0	91.6	93.8	94.8
duck	77.7	52.6	66.0	63.0	92.3	89.4	89.4	95.0
**egg**	97.1	99.2	100.0	99.9	99.8	97.3	99.8	100.0
**glue**	99.4	95.7	100.0	99.3	100.0	99.1	99.7	100.0
hole	52.8	82.0	49.0	71.8	92.1	89.3	93.2	95.2
iron	98.3	98.9	78.0	83.2	97.0	95.7	97.2	98.0
lamp	97.5	99.3	73.0	62.3	95.3	94.2	97.6	97.2
phone	87.7	92.4	79.0	78.8	92.8	93.7	96.5	97.2
MEAN	88.6	86.3	76.7	73.7	94.3	93.2	95.3	96.9

Bold objects indicate symmetrical objects.

**Table 3:** Quantitative evaluation of the 6D pose estimation (ADD-S) on the YCB-Video dataset. The object names in bold indicate symmetry.

	PoseCNN + ICP		Dense Fusion		YOLOPose V2		Ours (Inter)		Ours (Inter + Cross)		Ours (Inter + Cross + Weight)	
	AUC	<2 cm	AUC	<2 cm	AUC	<2 cm	AUC	<2 cm	AUC	<2 cm	AUC	<2 cm
master_chef_cam	95.8	100.0	95.2	100.0	91.3	-	94.3	100.0	95.6	100.0	96.1	100.0
cracker_box	92.7	91.6	92.5	99.3	86.8	-	92.2	95.3	93.8	98.5	95.2	99.6
sugar_box	98.2	100.0	95.1	100.00	92.6	-	98.7	100.0	99.4	100.0	100.0	100.0
tomato_soup_can	94.5	96.9	93.7	96.9	90.5	-	93.9	95.7	95.1	97.1	95.7	97.5
mustard_bottle	98.6	100.0	95.9	100.0	93.6	-	97.1	100.0	97.5	100.0	98.2	100.0
tuna_fish_can	97.1	100.0	94.9	100.0	94.3	-	94.8	99.0	96.6	100.0	98.4	100.0
pudding_box	97.9	100.0	94.7	100.0	92.3	-	93.3	93.9	97.6	100.0	98.3	100.0
gelatin_box	98.8	100.0	95.8	100.0	90.1	-	99.1	100.0	99.3	100.0	99.4	100.0
potted_meat_can	92.7	93.6	90.1	93.1	85.8	-	91.6	90.8	92.9	92.3	93.7	94.6
banana	97.1	99.7	91.5	93.9	95.0	-	91.1	93.4	94.2	99.4	98.1	99.7
pitcher_base	97.8	100.0	94.6	100.0	93.6	-	95.2	100.0	96.8	100.0	97.1	100.0
bleach_cleanser	96.9	99.4	94.3	99.8	85.3	-	86.5	97.8	94.2	98.4	98.6	99.7
**bowl**	81.0	54.9	86.6	69.5	92.3	-	83.8	53.2	86.7	70.3	87.2	73.9
mug	95.0	99.8	95.5	100.0	84.9	-	90.5	98.2	93.8	99.1	95.1	99.5
power_drill	98.2	99.6	92.4	97.1	92.6	-	94.8	97.4	96.6	97.8	96.9	98.3
**wood_block**	87.6	80.2	85.5	93.4	84.3	-	84.3	81.3	86.1	83.2	88.7	83.9
scissors	91.7	95.6	96.4	100.0	93.3	-	92.8	94.7	95.6	96.3	97.4	99.4
large_marker	97.2	99.7	94.7	99.2	84.9	-	88.6	96.7	95.4	98.7	96.2	99.0
**large_clamp**	75.2	74.9	71.6	78.5	92.0	-	73.2	68.3	78.2	74.8	80.3	76.9
**extra_large_clamp**	64.4	48.8	69.0	69.5	88.9	-	62.3	62.5	67.4	65.7	74.7	67.4
**foam_brick**	97.2	100.0	92.4	100.0	90.7	-	93.3	99.6	95.4	100.0	96.8	100.0
MEAN	93.0	93.2	91.2	95.3	90.1	-	90.5	93.5	93.4	95.8	94.7	96.5

Bold objects indicate symmetrical objects.

**Table 4:** Comparison of the accuracy of our method with increasing random noise.

**Noise Range (mm)**	0.0	5.0	10.0	15.0	20.0
**Accuracy**	98.3%	96.3%	95.6%	95.1%	93.8%

## Data Availability

Both datasets used in this study are publicly available and can be downloaded in the cited paper. If it is difficult to download, you can also request them from the corresponding author.

## Abbreviations

The following abbreviations are used in this manuscript:

CBAM	convolutional block attention module
CFFM	cross-modality feature fusion module
CNNs	convolution neural networks
CSAM	sampling center self-attention module
CWTM	contribution weight training module
ECAM	efficient channel attention module
FPS	farthest point sampling
ICP	iterative closest point
KNN	*K* nearest neighbor
MLP	multilayer perceptron
RANSAC	random sample consensus
SENet	squeeze-and-excitation network
